# Non-Arteritic Anterior Ischemic Optic Neuropathy: Challenges for the Future

**DOI:** 10.3389/fopht.2022.848710

**Published:** 2022-03-15

**Authors:** Alison Gibbons, Amanda D. Henderson

**Affiliations:** ^1^ Wilmer Eye Institute, Johns Hopkins University School of Medicine, Baltimore, MD, United States; ^2^ Department of Neurology, Johns Hopkins University School of Medicine, Baltimore, MD, United States

**Keywords:** non-arteritic anterior ischemic optic neuropathy (NAION), neuroprotection, neuroregeneration, retinal ganglion cell (RGC), neuroinflammation

## Introduction

Non-arteritic anterior ischemic optic neuropathy (NAION) is the most common acute unilateral optic nerve (ON)-related cause of vision loss in people over age 50 (1, 2). However, despite the frequency with which this condition occurs, there is no treatment proven to improve vision in patients affected by NAION. Patient evaluation initially focuses on the exclusion of mimickers, specifically arteritic anterior ischemic optic neuropathy secondary to giant cell arteritis, as well as optic neuritis in atypical cases. After confirmation of the NAION diagnosis, the focus shifts to the identification of modifiable risk factors, including hypertension, diabetes, hyperlipidemia (3), obstructive sleep apnea (4, 5), and phosphodiesterase-5 inhibitor use (6, 7). Optimizing treatment of modifiable risk factors may decrease the risk of developing a sequential NAION in the fellow eye.

The pathophysiology of NAION, while not fully elucidated, is thought to be secondary to decreased perfusion of the anterior ON from short posterior ciliary arteries, leading to the development of optic disc swelling and, eventually, a compartment syndrome (8). The cause of the decreased perfusion (eg, hypotension, microthrombosis, a combination of these, or something else entirely) has not been confirmed. Studies also suggest that the inflammatory response to the initial injury may play a role in the resultant neuronal damage and visual loss (9, 10). Therefore, various potential neuroprotective and neuroregenerative treatments for NAION have been (and continue to be) evaluated.

## Clinical Research

Many potential agents and procedures have been clinically assessed in the treatment of NAION, but none have clearly shown benefit and there is no widely accepted treatment regimen.

### Medical Treatments

In a small double-masked, placebo-controlled study, no improvement in visual function was demonstrated with phenytoin treatment (11).

Aspirin has not shown benefit for visual outcome in eyes affected by NAION (12). While data have been inconsistent for a role in risk reduction for second eye involvement (13–15), there is no convincing evidence that aspirin prevents future NAION (16). Aspirin may be appropriate for secondary prevention of cardiovascular events (17), but the role of aspirin in primary prevention of cardiovascular events, even in the setting of known vasculopathic risk factors (and/or a prior NAION), is less clear, and recent reports have shown an increase in major hemorrhage without any significant reduction in risk (18). Therefore, the routine use of aspirin in patients with NAION is not recommended.

Both retrospective and prospective studies have evaluated the use of brimonidine, hypothesized to have neuroprotective potential, in patients with NAION. Neither study demonstrated benefit (19, 20).

The use of oral steroids in NAION is controversial. Hayreh and Zimmerman reported on a cohort of 696 eyes with NAION, comparing those treated with oral steroids with those not treated. Notably, the patients themselves selected their treatment group, with no randomization, masking, or placebo control. Among eyes with initial visual acuity of 20/70 or worse, treated within two weeks of onset, visual acuity and kinetic visual fields (assessed subjectively) were more likely to improve in the steroid-treated group than in the group that received no treatment (21). However, other studies (including randomized controlled trials and a meta-analysis) have found no significant benefit from treatment with oral steroids but have shown an increased risk of steroid-related complications (22–25). Therefore, routine use of oral steroids for treatment of NAION is not recommended.

The use of erythropoietin (administered intravitreally or intravenously) to treat NAION also is controversial. One interventional case series reported visual improvement in 55% of eyes treated with intravitreal erythropoietin, with a trend toward initial improvement followed by a gradual decline in vision thereafter (26). There was no control group, but the authors argued that the rate of visual improvement was superior to the rate of 39.5% previously reported in the natural history of NAION (27). One prospective study evaluating treatment with intravenous erythropoietin showed no effect on visual outcomes (23), though another randomized trial with a shorter inclusion window (within five days of vision loss, rather than 14 days) reported that 55% of patients treated with erythropoietin (versus 34% treated with steroid and 31% receiving placebo) gained three or more Snellen lines at the six-month follow up when compared with baseline (28), raising the question of whether erythropoietin could be useful in patients who present soon after vision loss.

A recent randomized, controlled trial evaluating treatment with subcutaneous RPh201 (an extract of gum mastic with possible immunomodulatory and neuroprotective effects) in patients with chronic NAION also was disappointing.

### Surgical/Procedural Treatments

The Ischemic Optic Neuropathy Decompression Trial reported that ON sheath fenestration was ineffective and might be harmful in NAION (27). Hyperbaric oxygen treatment has not shown convincing benefit in NAION (29). While intravitreal anti-vascular endothelial growth factor (VEGF) therapy, used widely for the treatment of ischemic conditions of the retina, initially was reported as a promising treatment for NAION (30), no benefit was demonstrated in a non-randomized controlled trial (31). Intravitreal administration of QPI-1007 (a small interference RNA designed to inhibit expression of caspase 2) (32) and G-CSF (33) also have not demonstrated benefit.

## Basic and Translational Research

While the lack of benefit demonstrated in recent clinical trials has been discouraging to patients and the physicians who treat them, progress is being made in the laboratory setting. Two models of NAION, a rodent and primate model (rNAION and pNAION, respectively), have been developed for research into the pathophysiology of the disease, as well as for preclinical treatment trials (34, 35). Both models use laser-induced reactive oxygen species to promote capillary vascular thrombosis without affecting larger vessels (10). The pathophysiology in rNAION and pNAION mirrors the clinical disorder in terms of optic disc edema, ON axon loss, isolated retinal ganglion cell (RGC) loss, and ON dysfunction (10). Further, similar to human NAION, rNAION expresses significant variability in its severity and expression across different subjects, despite consistency of the induction technique (36).

### Neuroprotection

The development of the rNAION and pNAION models has facilitated the assessment of a number of potential neuroprotective interventions. Many of these interventions function by suppressing the inflammatory response following RGC injury. Prostaglandin J2 (PGJ_2_), an anti-inflammatory prostaglandin synthesized following central nervous system ischemia, led to a reduction in clinical, electrophysiological, and histological damage when administered as a single intravitreal dose five hours after the induction of pNAION (37) and immediately after the induction of rNAION (38). Potentially synergistic combination therapies with PGJ_2_ are being explored, though up to this point, none have demonstrated efficacy beyond that of PGJ_2_ alone (39). Daily topical ocular delivery of trabodenoson, a selective adenosine A_1_ agonist, was shown to reduce ON edema and preserve RGCs in rNAION, when compared with vehicle (40). Further, intravitreal injection of ciliary neurotrophic factor (CNTF) was shown to promote RGC survival when administered one day after rNAION induction (41). Recent work has also found E212, a Rho kinase (ROCK) inhibitor, to have a neuroprotective effect when injected intravitreally immediately following rNAION. E212 was shown to suppress neuroinflammation and oxidative stress, as demonstrated by increased superoxide dismutase activity and decreased reactive oxygen species formation, leading to RGC preservation when compared with vehicle-treated eyes (42). In addition to topical or intravitreal treatment, alternative drug delivery methods have also been explored. Polyamidoamine dendrimer nanoparticles have been shown to selectively target ischemic ON lesions in both pNAION and rNAION, suggesting that nanoparticle-linked therapeutics may provide a targeted route for drug delivery directly to the affected tissue in the future (43).

### Neuroregeneration

Published studies using NAION-specific animal models have primarily focused on neuroprotective interventions, with the goal of preventing RGC loss after injury. However, ON regeneration, with the goal of restoring vision following RGC death, is an alternative approach to the treatment of NAION and other optic neuropathies. In 2013, the National Eye Institute (NEI) Audacious Goals Initiative (AGI) in Regenerative Medicine established the goal “to restore vision through regeneration of neurons and neural connections in the eye and visual system”, thus directing significant resources toward this aim (44). Replacement of RGCs holds strong potential for restoring vision loss due to optic neuropathy and has been studied primarily in reference to glaucoma. RGC transplantation to restore vision requires multiple complex steps, each with its own unique challenges, including establishing a source for the RGCs, delivering the RGCs, promoting their survival and correct localization within the retinal structure, forming dendritic connections within the retina and growing axons toward the ON and, ultimately, further posterior to synapse in the lateral geniculate nucleus in a retinotopic arrangement, and ensuring myelination of the axons (45). While a complete review of the research in this area is beyond the scope of this paper, we will briefly discuss some exciting research breakthroughs.

Human-derived RGCs have been produced from numerous lineages, thus allowing for further study of transplantation *in vivo* (45). Some studies have shown functional improvements following RGC transplantation, including light-evoked electrophysiological responses from donor RGCs (46) and documented improvements in visually guided behaviors in recipient animals (47). However, there are still many challenges, particularly with regard to low rates of RGC engraftment and survival following transplant (45). One substantial limitation to engraftment of RGCs from an intravitreal approach is the structural barrier of the internal limiting membrane (ILM) (48). However, recent work has shown that the use of proteolytic enzymes to disrupt of the ILM prior to transplant is associated with a profound increase in neurite ingrowth in the retina (49).

Additionally, recent work has demonstrated that both molecular signaling and the external application of electric fields can be used to direct the growth of RGC axons. One study showed that a combination of neural activation and elevation of the pro-cell growth pathway mammalian target of rapamycin (mTOR) led to RGC axons regenerating long distances and forming connections with their correct targets (50). Another group demonstrated that ectopic expression of the *Oct3*, *Sox2*, and *Klf4* genes (three of the Yamanaka factors that can trigger mature cells to revert to an immature state) in mouse RGCs restored youthful DNA methylation patterns, promoted axon regeneration after injury, and reversed vision loss in a mouse model of glaucoma (51). Additionally, the application of electric fields was shown to direct axon growth from RGCs toward the cathode (52). A combination of these approaches may be required to promote and direct long distance axonal growth (initially toward the ON in the retinal nerve fiber layer, then through the ON, optic chiasm, and optic tract, to ultimately synapse in the lateral geniculate nucleus, all while maintaining retinotopic organization) following transplantation of RGCs.

## Discussion

While recent clinical trials have not identified an effective NAION treatment, they have collected vast amounts of data from patients affected by NAION. Further analyses of these data likely will advance our understanding of the factors surrounding NAION and perhaps provide insight into the similarities (and differences) between NAION in humans and experimental NAION in the animal models with which we work, thus clarifying the ways in which we interpret our laboratory results.

Regarding potentially neuroprotective treatments in the setting recent NAION, one key remaining challenge is to identify treatments that are effective within a clinically relevant treatment window, as most patients affected by NAION present days to weeks after the onset of vision loss, rather than within hours. Therefore, a treatment for which clinical trial recruitment would be feasible, and that could be anticipated to provide benefit in clinical trials and beyond, would need to be effective within this longer time window, rather than only when administered before or immediately following the onset of NAION. It is possible, and perhaps probable, that this may require combination therapies to address different inflammatory mediators at different time points after the acute ON injury. Studies in this area are ongoing.

Regarding RGC transplantation and ON regeneration, while exciting progress is being made on the various aspects of this approach, much work remains to develop a process to make this treatment a reality for our patients. Significant progress has been made in this area since the establishment of the NEI’s Audacious Goal. Collaboration between teams of vision scientists working on the different steps of this process will continue to be of utmost importance moving forward.

Both neuroprotective and neuroregenerative approaches hold promise to provide treatments for our patients with currently untreatable NAION, as well as other optic neuropathies. Until treatments become available, clinicians must continue to focus on risk factor identification and management in patients with NAION.

## Author Contributions

AH contributed to conception and design of the work. AG and AH contributed to the data acquisition. AG and AH each drafted initial sections of the paper. All authors contributed to manuscript revision and approved the submitted version.

## Conflict of Interest

The authors declare that the research was conducted in the absence of any commercial or financial relationships that could be construed as a potential conflict of interest.

## Publisher’s Note

All claims expressed in this article are solely those of the authors and do not necessarily represent those of their affiliated organizations, or those of the publisher, the editors and the reviewers. Any product that may be evaluated in this article, or claim that may be made by its manufacturer, is not guaranteed or endorsed by the publisher.
